# Nutritional Intervention Incorporating Expedited 10 g Protein Counter (EP-10) to Improve the Albumin and Transferrin of Chronic Hemodialysis Patients

**DOI:** 10.5402/2013/396570

**Published:** 2012-10-22

**Authors:** Su-Lin Lim, Jamie Lye

**Affiliations:** ^1^Dietetics Department, National University Hospital, 5 Lower Kent Ridge Road, Main Building, Level 1, Singapore 119074; ^2^Institute of Health and Biomedical Innovation, School of Public Health, Queensland University of Technology, Victoria Park Road, Kelvin Grove, Brisbane, QLD 4059, Australia; ^3^Centre for Research in Pedagogy and Practice and Department of Physical Education and Sports Science, National Institute of Education, NIE05-03-40, 1 Nanyang Walk, Singapore 637616

## Abstract

*Objective*. The expedited 10 g protein counter (EP-10) is a quick and valid clinical tool for dietary protein quantification. This study aims to assess the clinical effectiveness of the EP-10 in improving serum albumin and transferrin in chronic hemodialysis patients. *Methods*. Forty-five patients with low serum albumin (<38 g/L) were enrolled in this study. Parameters measured included dry weight, height, dietary intake, and levels of serum albumin, transferrin, potassium, phosphate, and kinetic modeling *(Kt/V).* The nutritional intervention incorporated the EP-10 in two ways (1) to quantify protein intake of patients and (2) to educate patients to meet their protein requirements. Mean values of the nutritional parameters before and after intervention were compared using paired *t*-test. *Results*. Three months after nutritional intervention, mean albumin levels increased significantly from 32.2 ± 4.8 g/L to 37.0 ± 3.2 g/L (*P* < 0.001). Thirty-eight (84%) patients showed an increase in albumin levels, while two (4%) maintained their levels. Of the thirty-six (80%) patients with low transferrin levels (<200 mg/dL), 28 (78%) had an increase and two maintained their levels after intervention. Mean transferrin levels increased significantly from 169.4 ± 39.9 mg/dL to 180.9 ± 38.1 mg/dL (*P* < 0.05). *Conclusion*. Nutritional intervention incorporating the EP-10 method is able to make significant improvements to albumin and transferrin levels of chronic hemodialysis patients.

## 1. Introduction

Protein-energy malnutrition (PEM) is highly prevalent in chronic kidney disease (CKD) patients on hemodialysis (HD) [1–4]. Low serum albumin and transferrin are surrogate markers of PEM, with low serum albumin being one of the most commonly used in CKD [5].

The progressive deterioration of kidney function in CKD leads to retention of many substances that are normally excreted by the kidney [6]. The retention of uremic toxins and other comorbid conditions can lead to a lowered appetite and a decrease in protein and energy intake, which is often compounded by ill-advised imposition of various dietary restrictions [7]. 

Among the causes of malnutrition in HD patients, inadequate dietary protein intake appears to be one of the most common and important [8]. Low protein intake and low serum albumin have been shown to be independently associated with increased risk of mortality in patients on dialysis [9, 10]. Mortality increases significantly as albumin levels fall below 39 g/L [11]. In the United States, it has been projected that interventions to improve serum albumin by just 2 g/L in 50% of hypoalbuminemic dialysis patients could save 1,400 lives and decrease hospitalizations by 6,300 per year, although a causal relationship has not been established [12]. 

The National Kidney Foundation Kidney Disease Outcomes Quality Initiative (NKF KDOQI ) has proposed a minimum intake of 1.2 g protein per kilogram of body weight per day to ensure a neutral protein balance in the hemodialysis population [13]. Despite this recommendation, inadequate protein intake persists in many chronic HD patients [8, 14, 15]. Moreover, the persistent marked reduction of daily nutrient intake, even if limited to a single day of the week, has been shown to be an independent determinant of reversible impairment of nutritional status [15].

Studies have suggested that individual dietetic counseling has significant potential to decrease the incidence of PEM in HD patients [16, 17]. However, standardized approaches to counseling have not been well documented. Complicating the problem, increasing dietary protein intake brings about the associated concerns of hyperphosphatemia and hyperkalemia, as potential consequences of increased dietary phosphorus and potassium burden [7, 18, 19]. 

The EP-10 protein counter, a protein exchange list developed for the quantification and education of dietary protein intake during dietetic counseling, has recently been validated in the Asian setting [20]. The purpose of this study was to investigate the effectiveness of using the EP-10 in dietetic assessment and counseling to improve the albumin and transferrin levels of CKD patients on HD. 

## 2. Methods

### 2.1. Subjects

Forty-five patients on stable hemodialysis with serum albumin of less than 38 g/L were enrolled in the study. Forty of these patients were on phosphate binders prior to enrollment into the study. 

### 2.2. Data Collection

Data on age, sex, race, other relevant biochemical results, cause of renal disease, medications, years of dialysis, and comorbid conditions were collected at baseline from the patients' medical and clinical charts. Patients' dry weight and body mass index (BMI) were obtained from the same sources at baseline and at three months after intervention.

### 2.3. Biochemical Parameters

Serum albumin, serum transferrin, kinetic modeling (*Kt*/*V*), potassium, and phosphate levels were measured at baseline before nutritional intervention and at three months after intervention. Predialysis serum albumin levels were tested in the laboratory using bromocresol green colorimetric assay at wavelength of 630 nm. 

*Kt/V* was calculated using the following urea kinetic modeling formula [21, 22]:

(1) $$\begin{array}{c} \frac{Kt}{V}=-\ln\left(R-0.008*t\right)+\left(\left(4-3.5*R\right)*\frac{\text{UF}}{W}\right), \end{array}$$

where *R* is the ratio of postdialysis to predialysis serum urea nitrogen, *t* is time of dialysis in hours, UF is the amount of ultrafiltration (in liters), and *W* is the postdialysis weight (in kg).

### 2.4. Nutritional Intervention

Nutritional intervention was provided to patients by the same clinical dietitian (SLL) trained in renal nutrition. Areas covered included, but were not limited to, dietary recall, dietary protein intake quantification and assessment, and individualized dietary recommendations based on the eating habits of the patient. The latter two were done using the EP-10 method as described by Lim et al. (2012) [20, 23].

Patients were educated on the importance of adequate protein intake and the number of protein exchanges required using the EP-10 method. Recommended protein intakes were based on the NKF KDOQI recommendation of 1.2 g protein per kilogram, body weight per day [13]. These were then explained to the patient using food models and pictures of protein portions based on EP-10 protein exchanges. The EP-10 food exchange list and a written individualized meal plan that included protein distribution were also given to each patient. 

Patients whose usual dietary intake provided insufficient calories were educated on ways to increase their caloric intake. Patients assessed as being unable to meet their nutritional requirement through diet alone were prescribed suitable nutritional supplements.

In addition to education on protein and caloric requirements, dietary implications of metabolic disorders such as hyperphosphatemia, hyperkalemia, hyperlipidemia, and diabetes mellitus were addressed during the counseling session when necessary.

## 3. Statistical Analysis

All statistical analyses were performed using Statistical Package for Social Science (PASW Statistics, Rel. 18.0.1. 2009. Chicago: SPSS Inc.). The mean differences between the biochemical parameters at baseline and at three months postintervention were evaluated using the paired *t*-test when the normality assumption was satisfied. Where the latter was not satisfied, the Wilcoxon signed-rank test was used. Results were considered to be statistically significant at *P* < 0.05.

## 4. Results

### 4.1. Patient Demographics

Table 1 shows the mean age, age range, gender ratio, diabetic status, and mean duration of HD of the patients.

### 4.2. Nutritional Assessment and Anthropometric Parameters

Using the EP-10 method, the mean daily dietary protein intake of the study subjects was 45 ± 10 g. This was significantly lower than their mean protein requirement, which was 68 ± 9 g per day based on the 1.2 g protein/kg body weight per day as recommended by NKF KDOQI.

Of the forty-five patients, 31 (69%) reported no loss of appetite. Eight patients were underweight (BMI <20 kg/m^2^), 15 patients were of normal weight (BMI 20–24.9 kg/m^2^), and 22 patients were overweight or obese (BMI >25 kg/m^2^). 

Of the eight underweight patients, six showed an increase in dry weight of between 0.4 to 4.4 kg three months after intervention while the two remaining underweight patients maintained their dry weight. 

Seven patients from the overweight and obese group (BMI >25 kg/m^2^) had a decrease in weight between 0.5–3.7 kg. None of the patients from the overweight or normal weight group became underweight.

### 4.3. Biochemical Parameters

Table 2 shows the mean differences between the biochemical parameters at baseline and three months postnutritional intervention.

Thirty-eight (84%) patients showed a rise in albumin levels, while two (4%) patients maintained their albumin level. All eight underweight patients showed an increase in albumin levels between 1.0 to 13.0 g/L (mean increase of 6.6 ± 4.8 g/L) postnutritional intervention. Thirty-six (80%) patients had low transferrin levels (<200 mg/dL) initially. After nutritional intervention, 28 out of these 36 (78%) patients had an increase in transferrin levels and two maintained their levels. 

There was no significant rise in the mean levels of potassium, phosphate, and *Kt/V* (Table 2). None of the five patients who were not prescribed phosphate binders had a significant increase in serum phosphorus.

## 5. Discussion

Hemodialysis, amongst other renal replacement therapy, is an accepted and successful treatment modality for supporting patients with chronic renal failure. It plays a major role in extending the life expectancy of these patients. However, factors like malnutrition, age, and other comorbidities, with emphasis on cardiovascular disease, still cause serious complications and adversely affect quality of life and risk of mortality in this population [1–4, 20, 24].

Food exchange lists, primarily carbohydrate exchange lists, have been widely used for many years to facilitate meal planning in diabetes and weight reduction. However, there is a lack of literature showing that exchange lists improve understanding and dietary compliance. The results of this study show that the EP-10 protein counter, when incorporated into individualized dietetic counseling, can potentially improve the nutritional parameters of HD patients [20]. 

### 5.1. Serum Albumin and Transferrin

Both albumin and transferrin are hepatic transport proteins with transferrin having a shorter half-life than albumin. Both reflect protein status and are used extensively as markers of nutritional status. In a study by Neyra et al. (2000), it was found that changes in albumin can be reliably predicted by earlier changes in serum transferrin [25]. Decreases in serum albumin and/or transferrin can be caused by insufficient protein and/or caloric intake. These levels are also decreased in inflammation, as both albumin and transferrin are negative acute phase proteins. However, research has shown that albumin levels in HD patients can respond to nutritional interventions despite high levels of inflammatory markers [26].

Several studies have reported a strong association between hypoalbuminemia and morbidity and mortality rates in HD patients, suggesting that nutritional interventions that maintain or increase serum albumin to normal levels may be associated with improved long-term survival [1, 26–31]. Owen et al. (1993) found serum albumin to be the single most powerful predictor of patient survival, with a 2 g/L decrease in serum albumin levels associated with a 25% decrease in survival [27]. Given this strong association between hypoalbuminemia and patient outcomes, an intervention that successfully improves serum albumin levels may exert substantial effects on overall patient health and survival.

There are many barriers to adequate nutritional intake in HD patients, including poor nutrition knowledge, poor appetite, and other functional or lifestyle issues [26]. Targeting nutritional barriers has been shown to increase albumin levels, even in the presence of elevated inflammatory markers [26]. In our study, insufficient protein and/or energy intake did not appear to be a consequence of poor appetite, as the majority of our hypoalbuminemic patients reported having a normal appetite. The significant improvement in albumin and transferrin levels of patients in this study after nutritional intervention suggests that the EP-10 protein counter can be easily understood and used by patients to increase their protein intake and thus overcomes at least one barrier to nutritional adequacy.

### 5.2. Serum Phosphorus, Potassium, and *Kt*/*V*

The kidney plays an important role in maintaining the homeostasis of phosphorus and potassium in the body. However, the renal mechanisms that ensure a balance of phosphorus and potassium are impaired in patients with kidney disease, leading to increased risk of hyperphosphatemia and hyperkalemia, both of which are associated with higher mortality in patients on HD [32, 33].

While limited evidence is available on the correlation between dietary protein intake and serum potassium, dietary phosphorus intake is strongly and positively correlated with dietary protein intake, and a high protein intake is often associated with the development of hyperphosphatemia [18]. Hyperphosphatemia may worsen hyperparathyroidism and renal osteodystrophy, promote vascular calcification and cardiovascular events, and increase mortality [34–37]. *In vivo* phosphorus is mostly bound to proteins and other intracellular, carbon-containing molecules [18]. Hence it is naturally found in foods that are rich in protein [18], and imposing dietary phosphorus restriction is often associated with a reduction in dietary protein intake, leading to an increased risk of PEM and mortality [38]. 

The results of this study show that using the EP-10 as a dietary assessment and educational tool to increase dietary protein intake to recommended levels did not cause an increase in serum phosphorous, potassium, or *Kt/V*. This suggests that patients are able to understand and utilize the EP-10 to meet their recommended protein intake and achieve an improvement in their albumin level yet avoid the associated complications of hyperphosphatemia and hyperkalemia. It also suggests that restricting dietary protein below the current NKF KDOQI guidelines in an attempt to manage serum phosphorous is not necessary with prescription of and compliance to phosphate binders. 

### 5.3. Limitations and Future Work

More research needs to be conducted to understand the reasons for insufficient energy and/or protein intake in CKD patients on HD. Amongst many possible reasons, it is important to determine if a relationship exists between a patient's degree of knowledge and understanding and their dietary compliance, as successful individual dietetic counseling relies on this relationship. As this study does not include a control group, future research should focus on a randomized control trial to assess the effectiveness of EP-10 versus conventional methods in the clinical setting.

### 5.4. Practical Applications

The results of this study show that using the EP-10 as an assessment and educational tool during nutritional counseling of hemodialysis patients has the potential to significantly improve albumin and transferrin levels.

## Figures and Tables

**Table 1:** Demographics of study participants (*n* = 45).

Age (mean + SD years)	57.5 + 12.0
Age range (years)	36−83
Gender (male : female)	24 : 21 (53% : 47%)
Diabetic status (yes : no)	18 : 27 (40% : 60%)
Mean duration of hemodialysis (months)	48.5 + 46.7
Ethnic distribution	
Chinese	22 (49%)
Malay	15 (33%)
Indian	7 (16%)
Others	1 (2%)

**Table 2:** Mean differences between the biochemical parameters at baseline and three months after nutritional intervention (*n* = 45).

Biochemical parameters	Baseline (0 months)	3 months after intervention	Mean difference ± SD	95% Confidence interval		*P* value
	Mean ± SD	Mean ± SD		Lower	Upper
Serum albumin (g/L)	32.2 ± 4.8	37.0 ± 3.2	4.8 ± 5.2	3.2	6.3	<0.001^†^
Serum transferrin (mg/dL)	169.4 ± 39.9	180.9 ± 38.1	11.5 ± 33.5	1.4	21.5	0.027^†^
Potassium (mmol/L)	5.0 ± 0.9	5.1 ± 0.9	0.1 ± 1.0	−0.2	0.4	0.664
Phosphate (mmol/L)	2.1 ± 0.7	2.1 ± 0.07	0.0 ± 0.7	−0.2	0.2	0.887
*Kt*/*V*	1.0 ± 0.5	1.2 ± 0.4	0.1 ± 0.6	0.0	0.3	0.136

^†^Statistically significant (*P* < 0.05).
